# Diagnostic Value and Predictive Factors for a Positive Labial Minor Salivary Gland Biopsy for Sjögren’s Syndrome in a Tunisian Population

**DOI:** 10.31138/mjr.080724.hba

**Published:** 2025-06-30

**Authors:** Dhouha Bacha, Mourad Touati, Zeineb Meddeb, Ahlem Lahmar, Salwa Hamzaoui, Sana Ben Slama

**Affiliations:** 1 Pathology Department;; 2 Internal Medicine Department; University Hospital Centre Mongi Slim, La Marsa Tunisia, Faculty of Medicine of Tunis, Tunis El Manar University, Tunis, Tunisia

**Keywords:** reliability, minor salivary gland, histology, diagnosis, Sjögren’s syndrome

## Abstract

**Objectives:**

To examine the performance of the minor salivary gland biopsy (MSGB) to diagnose Sjögren’s syndrome (SS) and to identify predictive factors for MSGB’s positivity in Tunisian SS-suspected patients.

**Methods:**

In a retrospective study, histopathological evaluation of MSGB from SS suspected- patients were examined. The classifications of the American-European Consensus Group (AECG, 2002) and the American College of Rheumatology/European League Against Rheumatism (ACR/EULAR, 2016) have been applied. We classified a positive MSGB when a focus score ≥ 1 and/or Chisholm and Mason grading ≥ 3 were observed. The sensitivity, specificity, positive predictive value (PPV), and negative predictive value (NPV) of MSGB were assessed, and the area under the ROC curve was performed to evaluate its diagnostic accuracy.

**Results:**

One hundred and two MSGBs were examined. They were positive in 48 patients (47%). For the positive diagnosis of SS, MSGB had 77,6% sensitivity, 93,2% specificity, 93,8% PPV and 75,9% NPV. With an air under the curve (AUC) of 0.854, MSGB was considered an excellent discriminating test in SS diagnosis. Keratoconjunctivitis sicca (p=0.04), elevated erythrocyte sedimentation rate (p=0.036), leukopenia (p=0.025), positive antibodies: anti-Ro/SSA (p=0.029), anti-Ro/SSA, anti-La/SSB (p=0.037), antinuclear (p=0.01), anti-extractable nuclear antigen (p=0.04), positive rheumatoid factor (p=0.032), positive elevated IgG levels (p=0.03) and abnormal unstimulated whole salivary flow rate (p=0.002) were predictive of a positive MSGB.

**Conclusion:**

In cases of suspected SS, a predictive scoring system incorporating these clinical and biological factors will streamline MSGB indications and serve as a diagnostic tool for positive SS diagnosis in research studies.

## INTRODUCTION

Sjögren’s syndrome (SS) is an autoimmune exocrinopathy characterised by the combination of xerostomia, xerophthalmia, and extra-glandular immune-inflammatory systemic manifestations.^1^ This sicca syndrome, results from lymphocytic inflammatory infiltration of the salivary and lacrimal glands and ranks as the second most common connective tissue disorder worldwide after systemic lupus erythematosus.^2^

SS can be primary when it occurs in the absence of another underlying in isolation or secondary to another connective tissue disorder or autoimmune disease. Currently, there is no simple, specific, and singular diagnostic test. A positive diagnosis relies on a comprehensive evaluation of anamnestic, clinical, biological, and histological evidence. Moreover, due to the clinical polymorphism of the disease, diagnosis can sometimes be delayed. Various classifications, incorporating diagnostic criteria for SS, have been proposed, with the most widely used being the American-European Consensus Group (AECG) 2002 criteria and more recently the American College of Rheumatology / European League Against Rheumatism (ACR/EULAR) 2016 criteria.^3,4^

Among the items of these criteria, the minor salivary gland biopsy (MSGB) enables a semi-quantitative histopathological assessment of lymphocytic inflammatory infiltrates based on the Chisholm and Mason grading score and the focus score (FS).^5^

Historically considered the gold standard for SS diagnosis, MSGB has nonetheless attracted attention from various studies regarding its diagnostic yield, yielding mixed results.^6^

Several studies have sought to identify predictive factors for a positive MSGB, aiming to limit potentially unnecessary indications. However, there is limited consensus among their conclusions.^7,8^ In this context, we conducted this study to identify associations of clinical and/or biological criteria, predictive of a positive MSGB for SS.

## MATERIALS AND METHODS

### Study design

This is a retrospective, descriptive, and monocentric study over a period of 6 years from January 2017 to January 2023. It was based on the review of slides from the MSGB prescribed by the physicians of the Internal Medicine Department of the same hospital for patients investigated for suspected SS.

### Study population

Our study included the MSGBs of patients in whom SS was initially suspected based on clinical and/or biological data. SS was diagnosed according to the ACR/EULAR 2016 criteria,^4^ for primary SS and those of the AECG 2002 criteria,^3^ for secondary SS.

We did not include patients with a history of radiation treatment to the head and neck, active hepatitis C infection (confirmed by polymerase chain reaction), Acquired immunodeficiency syndrome, Sarcoidosis, Amyloidosis, Graft-versus-host disease and IgG4-related disease. We also excluded patients whose MSGBs were non-contributory due to their small sizes or technical artifacts.

### Data collection / Diagnostic tests and clinical information

The data pertaining to various epidemiological, clinical, paraclinical, and histopathological aspects were collected from the patients’ medical records. Information gathered for each patient included age, gender, family and personal medical history, discovery circumstances, glandular and extra-glandular manifestations of SS, as well as biological data and conducted supplementary examinations.

The diagnosis of xerostomia was established either through patient interviews or by the presence of parotid enlargement, less commonly swelling of the submandibular glands, a depapillated or fissured tongue, and/or reduced unstimulated whole saliva (UWS) flow rate. The extra-glandular manifestations encompassed various involvements: joint, pleuro-pulmonary, renal, hematopoietic organs, neuropsychiatric, cardiac, cutaneous, muscular, digestive, and osseous. We also considered the presence or absence of general signs, namely constitutional symptoms defined by asthenia, anorexia, and/or weight loss exceeding 10 kg in 6 months, along with the presence of fever or night sweats. Involvement of one or more organs was determined based on functional symptoms reported by the patient and/or abnormalities identified during physical examination and/or various conducted supplementary examinations.

### Pathological study

Pathological data were collected based on the histological rereading of MSGB slides by the same pathologist (DB). For each case, we determined the number of fragments removed and the total surface area of the fragments in mm^2^ . These fragments were studied on multiple section levels.

The slides studied were stained with Haematoxylin and Eosin. Histological data included: number of lobules, composed of acini and ductal structures and type and abundance of inflammatory cells. These inflammatory cells (lymphocytes and histiocytes) were evaluated according to the Chisholm and Mason classification, by determining the number of foci (a focus = an aggregate of at least 50 lymphocytes or histiocytes, which may include a few plasma cells in the periphery). The focus score is reported as raw number of lymphocytic foci per 4 mm^2^ .

In total, the conclusions of the histological reports followed Chisholm and Mason’s classification and Focus Score (**Table 1**).

**Table 1.** Chisholm and Mason classification and Focus Score.

	**Grade**	**Infiltrate density**	**Focus Score**
**Normal MSGB**	Grade 0	Lobules of normal morphology and absence of inflammatory infiltrate	0
**Chronic lymphocytic sialadenitis**	Grade 1	Light inflammatory infiltrate limited to a few scattered cells	0
	Grade 2	Moderate inflammatory infiltrate but less than one focus	0
	Grade 3	One focus	1
	Grade 4	More than one focus	>1

MSGBs were considered inconclusive with the diagnosis of SS for grades 0,1 and 2 (negative MSGB) and concordant with the diagnosis of SS (positive MSGB) for grades 3 and 4.

### Statistical analysis

Data were entered and analysed using SPSS®. Proportions were presented with a 95% confidence interval. Continuous quantitative variables were illustrated by mean and standard deviation, and qualitative variables by percentage. Means were compared using Student’s t-test. The search for an association between a positive MSGB and several clinical and para-clinical criteria was carried out using Pearson’s Chi-2 test. Where the theoretical number of patients was less than 5, a correction was made using the Fisher test.

The search for these criteria was carried out using univariate analysis (factor by factor).

To identify predictive factors of a positive MSGB, we performed a multivariate analysis using logistic regression. This method involved the introduction of all factors whose “p” significance levels were < 0.2 in univariate analysis.

These criteria were: age, gender, various personal histories, smoking habits, various glandular and extra-glandular manifestations, general signs, Raynaud’s phenomenon, presence or absence of serum protein abnormalities (elevated erythrocyte sedimentation rate (ESR), elevated C-Reactive Protein (CRP), polyclonal hypergammaglobulinemia), hemogram abnormalities (leukopenia, lymphopenia), positive autoantibodies (rheumatoid factor, anti-nuclear, anti-SSA, anti-SSB, anti-ENA), immunoglobulin abnormalities (elevated IgG levels), pathological Schirmer’s test, pathological un-stimulated UWS flow rate and pathological Tear Break-Up Time (TBUT).

The sensitivity, specificity, positive predictive value (PPV) and negative predictive value (NPV) of MSGB were calculated. The diagnostic accuracy of MSGB was assessed using the receiver operating characteristic (ROC) curve, by calculating the area under the curve (AUC). In all statistical tests, the significance level was set at 0.05.

## RESULTS

### Study characteristics

Our series had interested 102 patients, managed at the internal medicine department of our hospital for a suspicion of SS, collated over a period of 6 years, from January 2017 to January 2023. The MSGBs prescribed for these patients were examined at the pathology department of the same hospital.

Our series included 7 men (6.9%) and 95 women (93.1%), with a sex-ratio (male/female) of 0.07. The average age of the patients was 55.45 years. First-degree relatives of 8 patients (7.8%) had a history of autoimmune disease.

The most frequent patient’s medical histories were: Type 2 diabetes in 19 patients (18.6%), hypertension in 16 patients (15.7%), dysthyroidism in 15 patients (14.7%), asthma in 10 patients (9.8%), and dyslipidaemia in 8 patients (7.8%). Six patients were smokers (5.9%). Three main discovery circumstances of SS were identified: glandular involvement was reported in 78 patients (76.5%), extra-glandular involvement was noted in 44 patients (43.1%), and biological signs were a revealing factor in 25 patients (24.5%).

### Clinical manifestations

General signs were present in 30 patients (29.4%), distributed as follows: Asthenia was reported in 26 patients (25.5%), weight loss was found in 19 patients (18.6%), anorexia in 14 patients (13.7%), fever in 18 patients (17.6%) and night sweats in 5 patients (4.9%). A subjective dry syndrome (xerostomia and/or xerophthalmia) was present in 83 patients (81.4%). Xerosis (dry skin) was present in 60 patients (58.8%). Keratoconjunctivitis sicca was present in 35 patients (34.3%). Parotidomegaly were noted in 12 patients (11.8%).

The distribution of patients according to extra-glandular manifestations is shown in **Table 2**.

**Table 2.** Distribution of patients according to extra-glandular manifestations.

**Extra-glandular manifestations**	**N**	**Prevalence**
**Articular**	71	69.6%
**Cutaneous**	60	58.8%
Raynaud's phenomenon	55	53.9%
**Pulmonary**	41	40.2%
**Neurological**	32	31.4%
**Digestive**	18	17.8%
**Haematopoietic organs**	17	16.7%
Splenomegaly	7	6.8%
Lymphadenopathy	14	13.7%
**Renal**	13	12.8%
**Osseous**	13	12.8%
**Muscular**	12	11.8%
**Cardiac**	8	7.8%
**Psychiatric**	8	7.8%

### Biological abnormalities

All the biological anomalies found in our series are summarised in Table 3.

**Table 3.** Distribution of patients according to biological abnormalities.

**Anomaly**	**N**	**Prevalence**
**Leukopenia**	38	37.3%
**Lymphopenia**	34	33.3%
**Anaemia**	26	25.5%
**Neutropenia**	7	6.9%
**Thrombopenia**	2	2%
**Elevated ESR**	57	55.9%
**Elevated CRP**	35	34.3%
**Polyclonal hypergammaglobulinemia**	38	37.2%
**Elevated IgG levels**	7	6.7%
**Positive RF**	46	45.1%
**Positive anti-Ro/SSA antibodies**	46	45.1%
**Positive anti-Ro/SSA + anti-La/SSB antibodies**	20	19.6%
**Positive ANA**	77	75.5%
**Positive anti-ENA antibodies**	56	54.9%

ESR: Erythrocyte sedimentation rate; CRP: C-Reactive Protein; ENA: Extractable nuclear antigen; RF: Rheumatoid factor; ANA: Antinuclear antibody; UWS: Unstimulated whole salivary; TBUT: Tear break-up time; SSA: Sjögren’s syndrome type A; SSB: Sjögren’s syndrome type B.

### Exploration of dry syndrome

During exploration of the dry syndrome, the TBUT was abnormal in 31 patients (30.4%), the Schirmer’s test was positive in 62 patients (60.8%), and UWS flow rate, performed in only 12 patients, was decreased in 9 (75%).

### Suspected and confirmed diagnoses of SS

Of the 102 patients included in our series, secondary SS was suspected in 27 patients (26.5%), while primary SS was suspected in 75 patients (73.5%). The diagnosis of primary SS was confirmed in 40 patients (53.3%), whereas secondary SS was confirmed in 18 patients (66.7%). The diagnosis of SS was therefore confirmed in 58 patients (56.9%).

**Table 4** summarises the distribution of patients according to suspicion and confirmation of SS.

**Table 4.** Distribution of patients according to suspicion and confirmation of Sjögren’s syndrome.

	**Suspected**		**Confirmed**	
**Sjögren’s syndrome**	N	Prevalence	N	Prevalence
**Primary**	75	73.5%	40	53.3%
**Secondary**	27	26.5%	18	66.7%
**Total**	102	100%	58	

### Pathological characteristics of MSGB

The average number of fragments removed was 2.24 (±1.1), with extremes ranging from 1 to 6. The average total surface area of the sampled fragments was 5.6 (±2.6) mm^2^, with extremes ranging from 1 to 15 mm^2^ . The mean number of lobules found was 6.5 (±2.5), with extremes ranging from 1 to 17.

**Figure 1.**
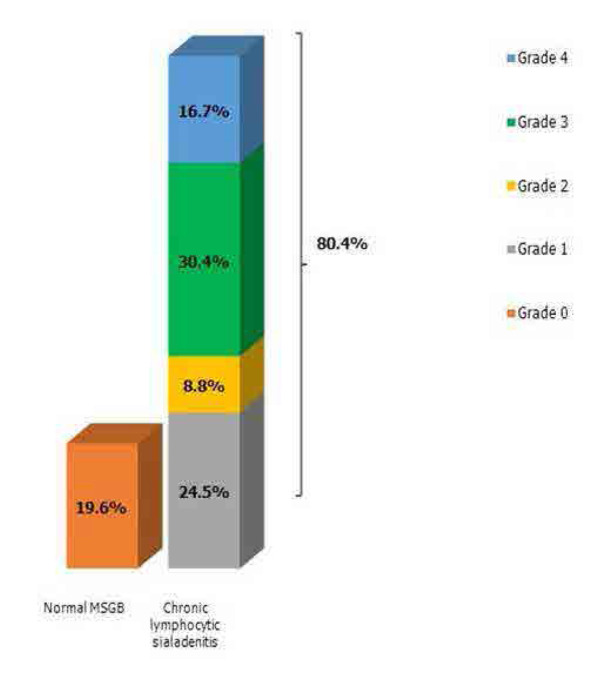
Distribution of patients according to the Chisholm and Mason classification.

**Figure 2.**
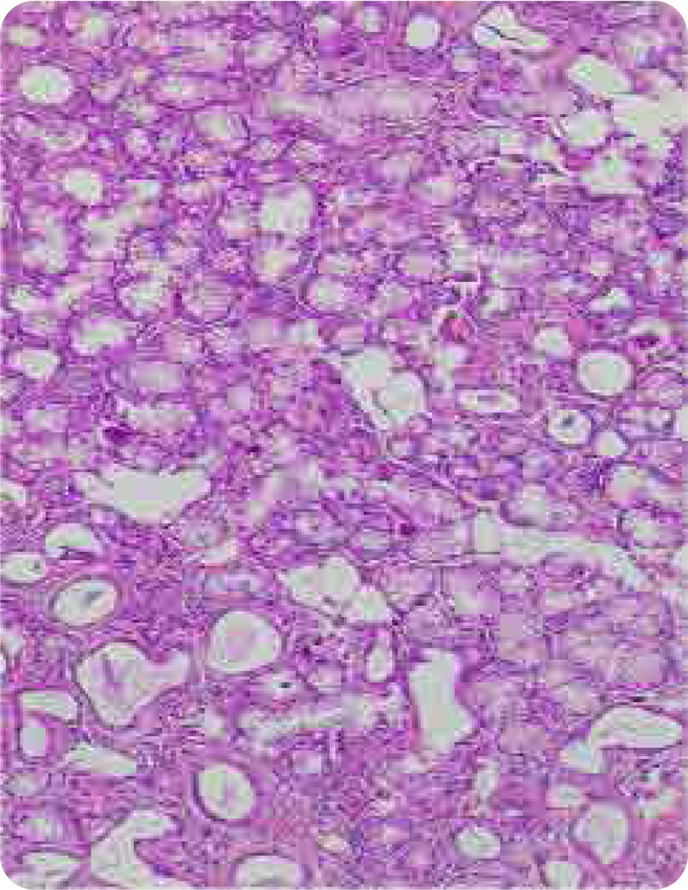
Normal salivary gland tissue.

The acini were atrophic in 19 patients (18.6%) and normal in 83 patients (81.4%). Ducts showed focal regression in 13 patients (12.7%), were dilated in 11 patients (10.8%) and normal in 78 patients (76.5%). The distribution of patients according to the Chisholm and Mason classification is shown in **Figure 1**. Normal salivary gland tissue is illustrated in **Figure 2**.

**Figure 3.**
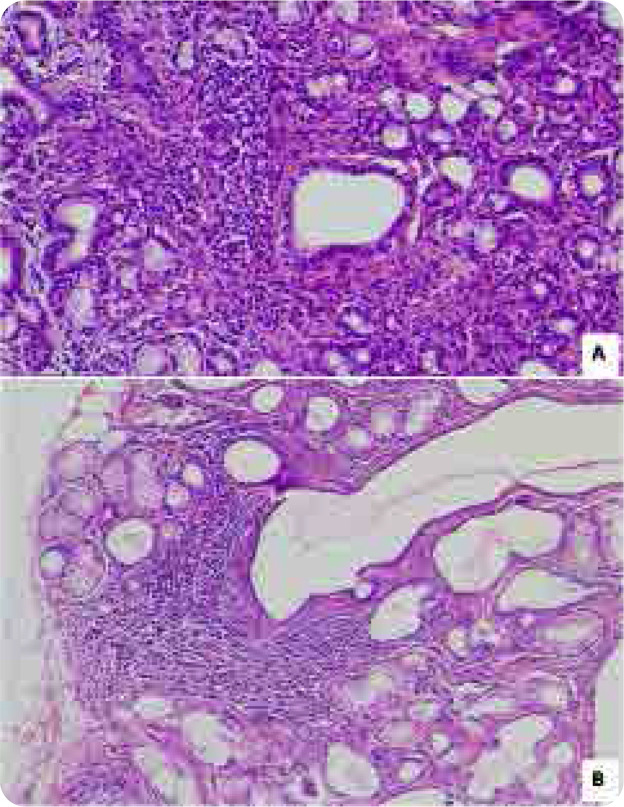
Grade 4 chronic lymphocytic sialadenitis from Chisholm and Mason, focus score >1. Multiple interstitial foci (100x) (A). One focus in the interstitial tissue (40x) (B)

MSGB was therefore positive (Focus Score ≥ 1) in 48 patients (47.1%) (**Figure 3**), while it was negative (Focus Score < 1) in 54 patients (52.9%).

### Diagnostic value of MSGB for SS

MSGB was positive in 48 patients (47.1%), 45 (93.7%) of whom had SS and was negative in 54 patients (52.9%), 13 (24%) of whom had SS according to 2016 ACR-EULAR criteria and/or the 2002 AECG criteria.

For the diagnosis of SS, the sensitivity of MSGB was 77.6% and its specificity was 93.2%. The PPV was 93.8% and the NPV was 75.9%. To evaluate the diagnostic value of MSGB in SS diagnosis, we plotted the ROC curve.

**Figure 4.**
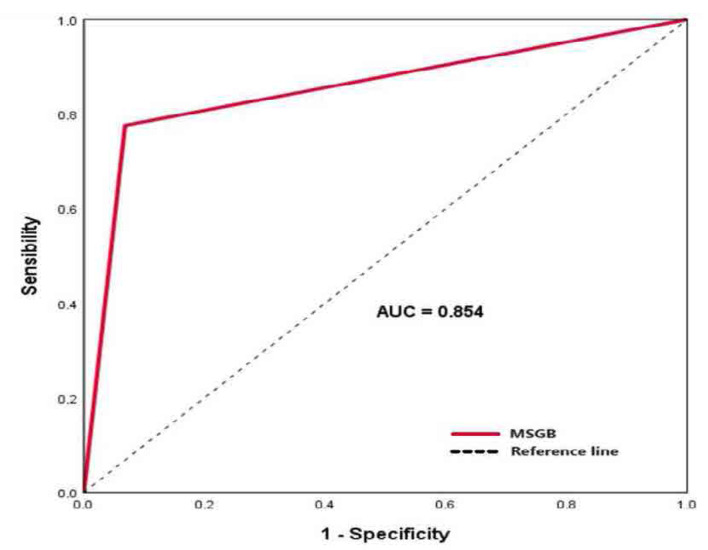
Evaluation of the diagnostic value of MSGB in SS diagnosis with an AUC of 0.854.

The results showed a good performance with an AUC of 0.854 (**Figure 4**).

The accuracy of Minor Salivary Gland Biopsy in diagnosing Sjögren’s syndrome was found to be quite similar for both primary and secondary forms of the disease. Specifically, the accuracy rates were 0.840 for primary SS and 0.884 for secondary SS.

### Correlations between a positive MSGB and various demographic, clinical and paraclinical criteria

We studied the various clinical and paraclinical factors that would be predictive of a positive MSGB. As summarised in **Table 5**, a large number of variables were positively associated with a positive MSGB. In univariate analysis, these variables were: parotidomegaly (p=0.039), keratoconjunctivitis sicca (p=0.021), Raynaud’s phenomenon (p=0.042), splenomegaly (p=0.034), elevated ESR (p=0.039), leukopenia (p=0.036), positive anti-Ro/SSA antibodies (p=0.03), double positivity for anti-Ro/SSA and anti-La/SSB (p=0.022), positive anti-ENA antibodies (p=0.02), positive RF (p=0.011), positive ANA (p=0.008), polyclonal hypergammaglobulinemia (p=0.032), elevated IgG levels (p=0.04), pathological Schirmer’s test (p=0.018) and decreased UWS flow rate (p=0.001).

**Table 5.** Variables positively associated with a positive MSGB.

**Variable**	**Negative MSGB**	**Positive MSGB**	**OR**	**95% CI**	**P**
**Age ≥ 55 years**	25	29	-	-	0.709

**Female**	52	43	-	-	0.181

**Type 2 diabetes**	9	10	-	-	0.590

**Hypertension**	6	10	-	-	0.178

**Dysthyroidism**	9	6	-	-	0.553

**Asthma**	6	4	-	-	0.638

**Dyslipidaemia**	5	3	-	-	0.573

**Smoking**	4	2	-	-	0.487

**Subjective dry syndrome**	44	39	-	-	0.785

**Keratoconjunctivitis sicca**	13	22	2.67	[1.15–60]	0.021

**Xerosis**	29	31	-	-	0.452

**Parotidomegaly**	3	9	3.92	[14–142]	0.039

**Fever**	11	7	-	-	0.444

**Weight loss**	10	9	-	-	0.976
**Articular manifestation**	36	35	-	-	0.493
**Pulmonary manifestation**	36	35	-	-	0.351
**Muscular manifestation**	7	5	-	-	0.690
**Renal manifestation**	8	5	-	-	0.506
**Neurological manifestation**	13	19	-	-	0.221
**Psychiatric manifestation**	3	6	-	-	0.217
**Digestive manifestation**	8	10	-	-	0.426
**Raynaud’s phenomenon**	24	31	2.8	[13-5.07]	0.042
**Osseous manifestation**	7	6	-	-	0.944
**Splenomegaly**	1	6	4.51	[1.03–13.52]	0.034
**Lymphadenopathy**	5	9	-	-	0.164
**Cardiac manifestation**	4	4	-	-	0.862
**Elevated ESR**	25	32	2.32	[1.04–5.18]	0.039
**Elevated CRP**	18	17	-	-	0.825
**Leukopenia**	15	23	2.39	[1.05–5.44]	0.036
**Lymphopenia**	20	14	-	-	0.4
**Polyclonal hypergammaglobulinemia**	15	23	2.29	[1.01–5.34]	0.032
**Positive anti-Ro/SSA antibodies**	17	29	3.32	[1.47–7.50]	0.03
**Positive anti-La/SSB antibodies**	12	17	-	-	0.140
**Positive anti-Ro/SSA + anti-La/SSB antibodies**	6	14	3.29	[1.15–9.4]	0.022
**Positive anti-ENA**	22	34	3.53	[1.54–8.06]	0.02
**Positive RF**	18	28	2.8	[1.25–6.26]	0.011
**Positive ANA**	35	42	3.8	[1.37–10.55]	0.008
**Elevated IgG levels**	0	7	2.98	[1.04–8.53]	0.04
**Pathological Schirmer’s test**	27	35	2.69	[1.17–6.17]	0.018
**Decreased UWS flow rate**	0	9	1.73	[1.28–3.34]	0.001
**Pathological TBUT**	11	20	-	-	0.72

OR: odds ratio; CI: confidence interval; ESR: Erythrocyte sedimentation rate; CRP: C-Reactive Protein; ENA: Extractable nuclear antigen; RF: Rheumatoid factor; ANA: Antinuclear antibody; UWS: Unstimulated whole salivary; TBUT: Tear break-up time; SSA: Sjögren’s syndrome type A; SSB: Sjögren’s syndrome type B.

In multivariate analysis, only the factors summarised in **Table 6** were predictive of a positive MSGB.

**Table 6.** Multivariate logistic regression analysis of factors associated with a positive MSGB.

**Variable**	**p**
**Parotidomegaly**	0.682
**Keratoconjunctivitis sicca**	**0.04**
**Raynaud’s phenomenon**	0.487
**Splenomegaly**	0.701
**Elevated ESR**	**0.036**
**Leukopenia**	**0.025**
**Polyclonal hypergammaglobulinemia**	0.062
**Positive anti-Ro/SSA antibodies**	**0.029**
**Positive anti-Ro/SSA + anti-La/SSB antibodies**	**0.037**
**Positive anti-ENA antibodies**	**0.04**
**Positive RF**	**0.032**
**Positive ANA**	**0.01**
**Elevated IgG levels**	**0.03**
**Pathological Schirmer’s test**	0.71
**Decreased UWS flow rate**	**0.002**

ESR: Erythrocyte sedimentation rate; CRP: C-Reactive Protein; ENA: Extractable nuclear antigen; RF: Rheumatoid factor; ANA: Antinuclear antibody; UWS: Unstimulated whole salivary; SSA: Sjögren’s syndrome type A; SSB: Sjögren’s syndrome type B.

## DISCUSSION

The diagnostic importance of MSGB in SS is well known, but recent studies investigating its true contribution in this context have found heterogeneous results.^6,9,10^ In order to optimise the indication for these biopsies, it is wise to rigorously determine the predictive factors leading to a positive MSGB. This reasoned approach aims to reduce the systematic and unnecessary prescription of MSGB, whatever the reason. The aim of our study was to describe the contribution of MSGB to the diagnosis of SS and to identify associations of clinical and/or paraclinical criteria predictive of a positive MSGB for this syndrome.

In the present study, we investigated the diagnostic value of MSGB assessment, performed with H&E staining in daily practice of the internal medicine department of our hospital. These biopsies concerned patients followed at this department with suspected SS. The MSGB slides were examined in the pathological department of the same hospital using two of the most common histological scores, the Chisholm and Mason grading system, and the FS.^11^

MSGB was positive in 48 patients (47.1%) and negative in 54 patients (52.9%).

We then calculated the various parameters assessing the diagnostic value of MSGB in the diagnosis of SS, namely: sensitivity, specificity, PPV, NPV and accuracy. These measures were 77.6%, 93.2%, 93.8%, 75.9% and 0.854 respectively. We also studied the epidemiological, clinical, and para-clinical data of these patients, and looked for those that were predictive of a positive MSGB.

In order to improve MSGB’s reliability in SS, standard-ising histopathological assessment should be an important objective for routine diagnosis. It is with this in mind that the Sjögren’s International Collaborative Clinical Alliance (SICCA) in collaboration with the Oxford University Centre for Evidence-Based Medicine have drawn up recommendations for the practice of MSGB. According to these guidelines, glandular tissue should include at least 4 lobules, and the minimum total surface area of biopsy fragments should be 8 mm^2^ . If the salivary glands are too small (<2 mm^2^ surface area), then 6 lobules should be harvested.^12,13^ Other studies have endorsed these recommendations and advocated sampling at least 4 to 6 lobules with a minimum surface area of 8 mm^2^ to allow adequate evaluation of the FS.^14^

### Performance of MSGB in the diagnosis of SS

The reproducibility of MSGB depends in part on pathologists and also on the sampler.^6^ It should be noted that in the elderly, it is common to have a FS ≥ 1 without SS.^15^ MSGB have good sensitivity (63.5% to 93.7%) and specificity (61.2% to 100%).^15^ They also have prognostic value, since a higher FS is associated with an increased risk of severe extra-glandular manifestations and lymphoma.^16^ The diagnostic performance of the MSGB varies between series, making it a poorly informative test according to the series by Wicheta et al.,^14^ and having a statistically significant and discriminating diagnostic power according to the series by Giovelli et al.,^17^ and Lee et al. (**Table 7**).^18^ In our series, the diagnostic performance of the MSGB was 0.854, making it an excellent discriminant test.

**Table 7.** Reliability of MSGB for the diagnosis of SS according to the main literature review.

**Authors**	**Sensibility**	**Specificity**	**PPV**	**NPV**	**Accuracy**
**Baeteman et al.**^10^ n=96	75%	100%	100%	90%	0,865
**Giovelli et al.**^17^ n=216	86,6%	97,4%	95%	92,6%	0,933
**Wicheta et al.**^19^ n=73	95,4%	76,4%	63,6%	97,5%	0,682
**Wicheta et al.**^9^ n=87	80%	87,5%	57,1%	95,5%	0,743
**Lee et al.**^18^ n=583	75,7%	90,7%	NR	NR	0,901
**Kutluk et al.**^20^ n=113	76,4%	95,1%	96,4%	69,6%	0,7
**Our series** n=102	77,6%	93,2%	93,8%	75,9%	0,854

### Predictive factors of a positive MSGB

Several reviews of the literature have analysed the various factors that would be predictive of a positive MSGB for the diagnosis of SS, with mixed results.

The results concerning subjective dry syndrome were heterogeneous: several series showed no correlation between the presence of a subjective dry syndrome and a positive MSGB, as did our study.^21–23^ Others have demonstrated this correlation.^24–25^

In our series, we found in univariate analysis that the presence of parotidomgaly was predictive of a positive MSGB. This result is in line with several studies.^26,27^ but not concordant with others.^22^ We also found in univariate analysis that keratoconjunctivitis sicca was predictive of a positive MSGB. This result concurs with that found by Brennan et al.^26^ The presence of splenomegaly was also predictive of a positive MSGB in univariate analysis in our study. This correlation was found in the series by Kilipiris et al. and Gerli et al.^28,29^

Raynaud’s phenomenon was also found to be predictive of a positive MSGB in our study. This was in line with the result found in other series. However, the study by Alhamad et al. found no correlation between the presence of this factor and a positive MSGB.^21^ In our study, the presence of an elevated ESR predicts a positive MSGB in a univariate study. This has also been demonstrated in the literature by Brennan et al.^26^

We also noted the presence of leukopenia as a predictive factor in univariate analysis. Other series have endorsed this result.^24,26–28^ However, the study by Brennan et al was inconsistent with our result.^26^

In a univariate study, we found that the presence of positive anti-SSA antibodies was a predictive factor. This result was unanimously supported by all studies in the literature.^17,21,22,24,26,27,30^

The positivity of both anti-SSA and anti-SSB antibodies was also predictive of a positive MSGB in our series. This result was consistent with that found in several studies.^17,24,27^

The presence of positive anti-ENA antibodies was a predictive factor in our study in univariate analysis. This was also found in the series by Conticini et al.^30^

We also noted in the univariate study that the presence of RF was predictive of a positive MSGB. This result was consistent with that found in a number of series.^24,26–28,30, 31^ In univariate analysis, we demonstrated that ANA positivity was also a predictive factor. This is in line with data from several studies.^21,22,24,26,27,32^

In univariate analysis, polyclonal hypergammaglobulinemia was found to be a predictive factor in our series. This result was found in other reviews of the literature.^24,27^ We also found in univariate analysis that an elevated IgG levels would predict a positive MSGB. This was in line with the literature.^26,28^

A pathological Schirmer’s test was also found to be predictive of a positive MSGB in our study. This was consistent with other studies.^17,25,26^

Decreased UWS flow rate was predictive of a positive MSGB in our series. This is in line with the literature.^17,24,26^ In our series, the predictive factor of a positive MSGB were the presence of keratoconjunctivitis sicca (p=0.04), which was consistent with the study by Brennan et al (OR: 6.80; IC95%: [1.96–5.23]; p=0.015).^26^ Other predictive factors were: elevated ESR (p=0.036), which was inconsistent with the study by Brennan et al.^26^, leukopenia (p=0.025), which was not found in the same study.^26^, positive anti-SSA antibodies (p=0.029), which was consistent with the study by Conticini et al. and Daniels et al.,^24,30^ positive anti-SSA and anti-SSB antibodies (p=0.037), as in the series by Daniels et al.^24^ We also found other predictive factors, such as positive anti-ENA antibodies (p=0.04), as in the study by Conticini et al.^30^, positive RF (p=0.032) and positive ANA (p=0.01), in line with the studies by Daniels et al and Conticini et al.^24,30^

Elevated IgG levels (p=0.03) and decreased UWS flow rate (p=0.002) were also predictive factors, as in the several series.^24,26,28^

In our study, there were some limitations. It focused on MSGBs prescribed by the internal medicine department, and did not include those prescribed by other departments, mainly rheumatology. This was due to a need to evaluate the MSGB requests performed by the department studied, which could subsequently modify their practices in this context. The Ocular Staining Score (OSS) (or Van Bijsterveld score), which is part of the ACR/EULAR 2016 criteria for the diagnosis of SS, was never performed in the hospital’s ophthalmology department in all patients. This parameter carries a diagnostic weight of 1 point. In some cases, the importance of this criterion lies in the fact that, if this test had been carried out and found positive, the diagnosis of SS in certain patients could have been made from the outset.

The anti-SSA antibodies studied were in the majority of cases anti-Ro52, whereas according to the ACR/EULAR 2016 criteria, it is anti-Ro60 that should be taken into account for the diagnosis of SS.

The results of our study have two implications: The first was to justify the standardised performance of MSGB according to best-practice recommendations, in order to guarantee a representative biopsy and thus improve diagnostic cost-effectiveness. The second implication is to justify the use of these predictive factors in daily practice for the indication of MSGB in suspected SS. In this way, patients with a low diagnostic suspicion will be spared and explored with other analyses.

To this end, the development of a clinico-biological score to predict a positive MSGB will facilitate these indications and serve as a tool for the positive diagnosis of SS in research studies. In addition, multicentre studies with larger sample sizes should be carried out in this context.

## CONCLUSION

MSGB has been a widely used test to diagnose SS. However, before ordering a lip biopsy, physicians must consider the entire clinical picture to confirm that this invasive test is truly necessary. It is in this context that the use of these predictive factors in day-to-day practice is of great interest for the indication of MSGB in cases of suspected SS.

## AUTHOR CONTRIBUTIONS

Dr Bacha: Conception and design, analysis and interpretation of data, pathologic study

Dr Touati: Drafting of the article, statistical analysis

Pr Hamzaoui: Critical revision of the manuscript for important intellectual content

Dr Meddeb: Acquisition, analysis and interpretation of data

Dr Ben Slama: Sourcing and editing of clinical images

Dr Lahmar: Final approval of the version to be published

All authors: Final approval of the version published, agreement to be accountable for the article and to ensure that all questions regarding the accuracy or integrity of the article are investigated and resolved.

## FUNDING

This research received no specific grant from any funding agency in the public, commercial, or not-for-profit sectors.
